# The complete chloroplast genome of *Vatica guangxiensis* S.L. Mo (Dipterocarpaceae): genome structure and evolution

**DOI:** 10.1080/23802359.2020.1835580

**Published:** 2020-11-13

**Authors:** Li-Li Deng, Hui-Zhen Qin, Yan-Cai Shi

**Affiliations:** Guangxi Institute of Botany, Guangxi Zhuang Autonomous Region and Chinese Academy of Sciences, Guilin, China

**Keywords:** Chloroplast genome, phylogenetic analysis, Dipterocarpaceae, *Vatica guangxiensis*

## Abstract

*Vatica guangxiensis* S.L. Mo is an evergreen large tree of Dipterocarpaceae. Herein, we assembled the complete chloroplast genome of *Vatica guangxiensis* by next-generation sequencing technologies. The complete chloroplast genome sequence of *Vatica guangxiensis* is 151,010 base pairs (bp) in length, including a pair of inverted repeat regions (IRs, 23,827 bp), one large single-copy region (LSC, 83,353 bp), one small single-copy region (SSC, 20,003 bp). Besides, the complete chloroplast genome contains 123 genes in total, including 83 protein-coding genes, 36 tRNA genes, and 8 rRNA genes. Phylogenetic analysis showed that *Vatica guangxiensis* has the closest relationship with *Vatica mangachapoi*. Our study lay a foundation for further research of *Vatica mangachapoi*.

*Vatica guangxiensis* is a characteristic tree species of tropical rain forest in Southern Yunnan of China and it is also an important timber tree species (Pachynocarpus and Retinodendron 2007). This species has limited number of individuals, only with three natural populations distributed in Southern Yunnan and Liushaoshan in Napo County of Guangxi, China. It was listed as an endangered plant species in China (Li et al. 2002). Mass extinction that is primarily due to large-scale habitat destruction caused by human activities. The best remedy to prevent extinction is habitat preservation. With the over-excavation of *Vatica guangxiensis*, their habitats have been broken up and the wild resources have decreased significantly over the last decades. Therefore, the *Vatica guangxiensis* is listed as an endangered species in the China Species Red List (Wang and Xie 2004). Therefore, we report the complete chloroplast genome of *Vatica guangxiensis*, in order to better understand the relationship between *Vatica guangxiensis* and related genera, and contribute to the effective conservation strategy of *Vatica guangxiensis*.

The sample of *Vatica guangxiensis*. was collected from Yachang Orchidaceae National Nature Reserve, Guangxi, China (24°44′N, 106°15′E) and the voucher specimen deposited at Herbarium of Guangxi Institute of Botany, Guangxi Zhuang Autonomous Region and Chinese Academy of Sciences (specimen code *Guangxiensis* _ GX).

High-quality genomic DNA of *Vatica guangxiensis* was extracted from leaves by TIANGEN plant genomic DNA kit, and sequenced by the BGISEQ-500 platform. With the chloroplast genome of *Vatica mangachapoi* (GenBank accession number NC_041485) as the reference sequences, we assembled the complete chloroplast genome from the clean reads by the GetOrganelle pipe-line (Jin et al. 2018), and then annotated the new sequences using the Geneious R11.15 (Kearse et al. 2012). Finally, a complete chloroplast genome of *Vatica guangxiensis* was obtained and submitted to Genbank (accession number MT934442).

The complete chloroplast genome sequence of *Vatica guangxiensis* is 151,010 base pairs (bp) in length, including a pair of inverted repeat regions (IRs, 23,827 bp), one large single-copy region (LSC, 83,353 bp), one small single-copy region (SSC, 20,003 bp). Besides, the complete chloroplast genome contains 123 genes in total, including 83 protein-coding genes, 36 tRNA genes, and 8 rRNA genes. In addition, the overall GC content of the genome was 37.2%.

In order to confirm the phylogenetic position of *Vatica guangxiensis*, a maximum likelihood analysis was performed by MEGA 6.0 (Tamura et al. 2013) with 1000 bootstrap replicates (Minh et al. 2013; Chernomor et al. 2016) based on 21 complete chloroplast genomes. All sequences were aligned with the HomBlock pipeline (Bi et al. 2018) and subsequently checked manually in Bioedit v5.0.9 (Hall 1999). The results showed that *Vatica guangxiensis* was sister to *Vatica mangachapoi* with 100% bootstrap support (Figure 1).

**Figure 1.**
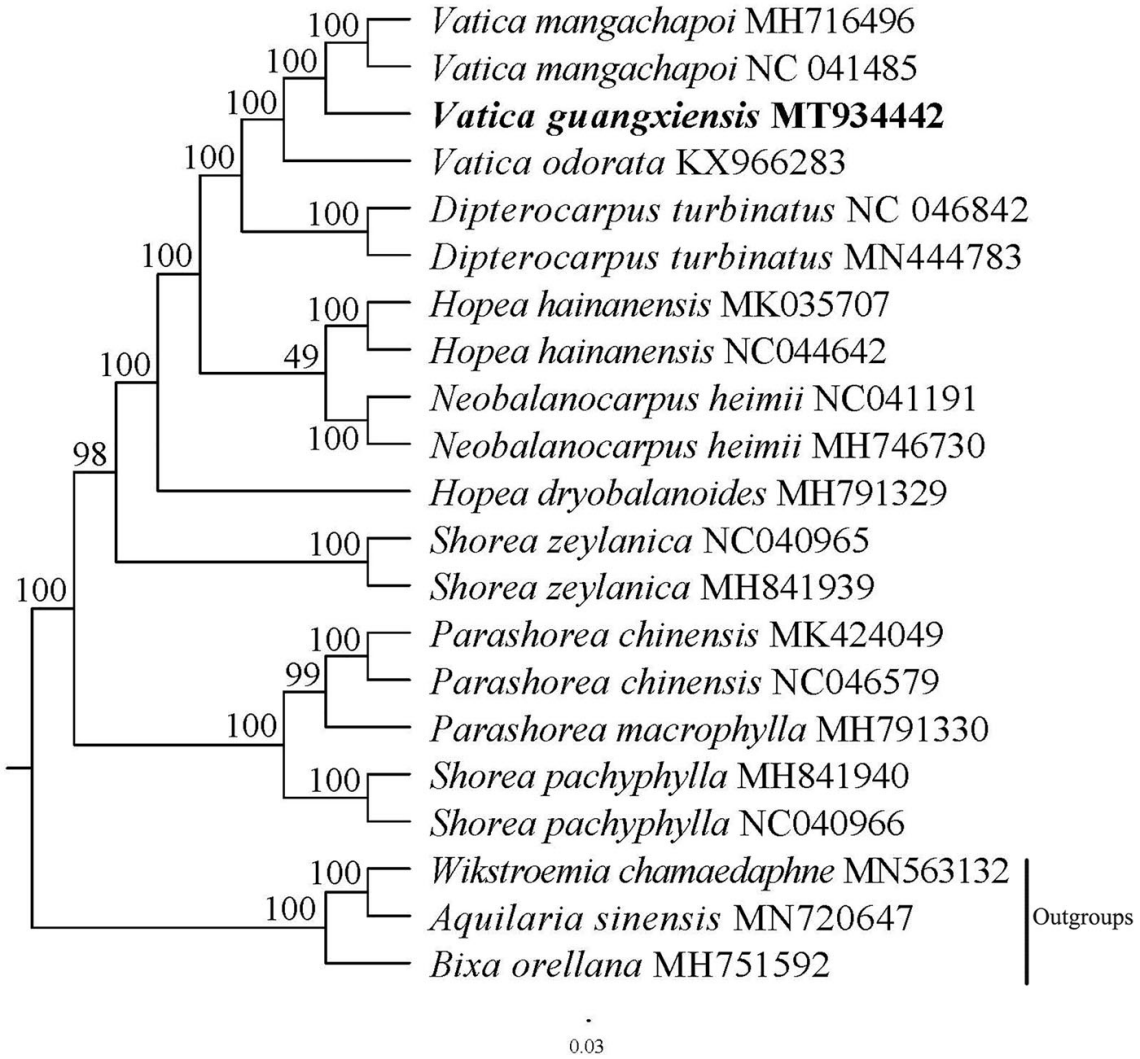
A Phylogenetic tree was constructed based on 21 complete chloroplast genome sequences. All the sequences were downloaded from NCBI GenBank.0.

## Data Availability

Data openly available in a public repository that does not issue DOIs. The data that support the findings of this study are openly available in [National Center for Biotechnology Information] at [https://www.ncbi.nlm.nih.gov/], reference number [MT934442].
